# A potential research target for cardiac rehabilitation: brain-derived neurotrophic factor

**DOI:** 10.3389/fcvm.2024.1348645

**Published:** 2024-04-19

**Authors:** Jianpeng Zou, Shijie Hao

**Affiliations:** ^1^ Department of Rehabilitation and Physiotherapy, Affiliated Hospital of Shandong University of Traditional Chinese Medicine, Jinan, China; ^2^ College of Rehabilitation Medicine, Shandong University of Traditional Chinese Medicine, Jinan, China

**Keywords:** brain-derived neurotrophic factor, cardiac rehabilitation, exercise, cardiovascular disease, brain-heart axis

## Abstract

Cardiovascular diseases pose a major threat to human life, functional activity, and quality of life. Once the disease is present, patients can experience varying degrees of problems or limitations on three levels: physical, psychological, and social. Patients with cardiovascular disease are always at risk for adverse cardiac events, decreased physical activity, psychoemotional disturbances, and limited social participation due to their varying pathologies. Therefore, personalized cardiac rehabilitation is of great significance in improving patients’ physical and mental functions, controlling disease progression, and preventing deterioration. There is a consensus on the benefits of cardiac rehabilitation in improving patients’ quality of life, enhancing functional activity, and reducing mortality. As an important part of cardiac rehabilitation, Exercise plays an irreplaceable role. Aerobic exercise, resistance training, flexibility training, and other forms of exercise are recommended by many experts. Improvements in exercise tolerance, lipid metabolism, cardiac function, and psychological aspects of the patients were evident with appropriate exercise interventions based on a comprehensive assessment. Further studies have found that brain-derived neurotrophic factor may be an important mediator of exercise's ability to improve cardiovascular health. Brain-derived neurotrophic factor exerts multiple biological effects on the cardiovascular system. This article provides another perspective on the cardiac effects of exercise and further looks at the prospects for the use of brain-derived neurotrophic factor in cardiac rehabilitation. Meanwhile, the new idea that brain-derived neurotrophic factor is a key mediator connecting the brain-cardiac axis is proposed in light of the current research progress, to provide new ideas for clinical rehabilitation and scientific research.

## 1 Introduction

In the worldwide, Cardiovascular diseases pose a significant risk to human health (1). Despite the great strides made in medical treatment, the adverse effects of cardiovascular disease on patients are persistent. As a result, patients often experience limitations in activities of daily living, reduced quality of life, and various psychological problems, further affecting social participation. Since cardiovascular diseases can lead to many of these physical and mental dysfunctions, rehabilitation is especially important. A cohort study showed that cardiac rehabilitation reduced the risk of all-cause mortality in patients with cardiovascular disease by 32% (2). Cardiac rehabilitation integrates interventions such as exercise, education, and behavior modification (3). Of these, exercise plays an important role in cardiac rehabilitation. Individualized, appropriate exercise prescriptions can maintain patients’ heart health, reduce the incidence of cardiovascular events, increase their mobility, and improve their quality of life (4).

Brain-derived neurotrophic factor(BDNF) is a member of the neurotrophic factor family and plays an important role in the nervous system. BDNF is involved in neuronal growth and development functional maintenance, and regulation of neuroplasticity (5). BDNF is mainly synthesized by neurons and glial cells (6).In the nervous system, BDNF is mainly distributed in the cerebral cortex, cerebellum, hippocampus, and other key areas (7). BDNF plays a role in several aspects such as recovery from brain injury after stroke, regulation of mood disorders, and delaying the progression of neurodegenerative diseases (8, 9). The potential of BDNF in cardiac rehabilitation has attracted scholarly attention in recent years (10). BDNF can cross the blood-brain barrier and enter the circulation. Relevant studies have shown that 70%–80% of BDNF in the blood circulation comes from the brain (11). Through blood circulation, BDNF is widely distributed in the human body, and its presence is found in organs such as the heart, thymus, and spleen (12, 13). BDNF can exert a variety of biological effects and can be expressed in a variety of cells, including cardiomyocytes (14), vascular endothelial cells (15), vascular smooth muscle cells (16), immune cells (17), and epithelial cells (18). In the cardiovascular system, BDNF is involved in the maintenance of vascular endothelial homeostasis, and it can also activate the corresponding signaling pathways through binding with tropomyosin-related kinase receptor B (TrkB) to play multiple roles, which is of great significance in cardiovascular injury repair and disease recovery (19). Considering the important role of BDNF in cardiovascular system health and disease rehabilitation, combined with the relevant research results of exercise promoting BDNF release in recent years, this paper proposes the view that exercise regulating BDNF level participation in cardiac rehabilitation, to open up a research perspective for cardiac rehabilitation.

## 2 Role of BDNF in the cardiovascular system

### 2.1 BDNF is a regulator of homeostasis in the cardiovascular system

BDNF is an important regulator of cardiovascular system homeostasis. BDNF is involved in maintaining vascular endothelial integrity and plays an important role in maintaining cardiovascular stability (15). By recruiting vascular endothelial cells and upregulating prosurvival factors, BDNF may exert cardioprotective effects (20). In addition, BDNF can promote M2 macrophage polarization through the inhibition of the STAT3 pathway and thus play an anti-atherosclerotic role (21). Once the level of BDNF in the blood circulation is reduced, it can affect its protective effect on the cardiovascular system, which may lead to the occurrence of disease.Taking coronary heart disease as an example, the diminished cardioprotective effects of BDNF may be an important reason for the increased incidence of coronary heart disease in the elderly. Coronary heart disease is based on the pathology of coronary atherosclerosis and is characterized by an imbalance between coronary blood supply and myocardial demand. As a chronic disease, coronary heart disease (CHD) has become an important public health problem worldwide (22). There is a close association between BDNF and coronary heart disease. Clinical studies have found reduced plasma BDNF concentrations in patients with coronary artery disease (23). BDNF has anti-atherosclerotic and vascular endothelial protection effects, and its decreased secretion directly affects cardiovascular health. In addition, BDNF is involved in substance metabolism and plays a role in the control of coronary heart disease risk factors.BDNF is involved in lipid metabolism and may regulate atherosclerotic plaque formation as well as vascular endothelial inflammatory processes. An animal study showed that BDNF enhanced lipid metabolism and aerobic glycolysis in rats (24). The reduction of BDNF levels can affect the metabolism of related substances, leading to the emergence of obesity, elevated blood glucose, etc., and adversely affecting patients with coronary heart disease (25). Based on the analysis of the above reasons, the reduction of circulating BDNF levels may be an important reason for the development of cardiovascular diseases in elderly patients (26, 27). Relevant studies have confirmed that the basal level of plasma BDNF is reduced in the elderly (28). Since most of the BDNF in plasma originates from the brain, decreased neurologic function may be the main reason for the decrease in circulating BDNF (12, 29). With the process of physiological aging, the elderly will experience insufficient cerebral perfusion and microcirculatory disorders, reduction in the volume of gray and white matter as well as diminished activity or even death of some neurons. All of the above internal factors combined with external stimuli such as stress and lack of activity can lead to a significant decrease in BDNF expression. Thus, reduced BDNF secretion due to brain hypoplasia, which in turn leads to altered cardiovascular homeostasis, may be one of the pathogenic mechanisms of coronary heart disease.

### 2.2 BDNF is a functional protein involved in myocardial endogenous repair

BDNF plays a key role in maintaining the structural and functional integrity of the heart, myocardial injury repair, and regeneration (30). As an important functional protein, BDNF plays a role in acute and chronic cardiovascular injury. Under myocardial ischemia and hypoxia, inflammatory factor stimulation, and other conditions, the expression of BDNF increases, which can further promote neoangiogenesis, and at the same time can play a role in anti-apoptosis of cardiomyocytes and regulation of myocardial contractility (19). The above cardiac effects of BDNF are mainly realized through its binding to tropomyosin-related kinase receptor B (TrkB)). The combination of BDNF and tropomyosin-related kinase receptor B can activate signaling pathways such as PLCγ, PKCα, and HIF-1α to increase the expression of VEGF, thereby promoting angiogenesis (31); At the same time, through certain mechanisms, it can inhibit oxidative stress and inflammatory response, and enhance cardiac function (32). BDNF is involved in the process of cardiac structural and functional recovery during the acute and recovery phases of myocardial infarction. BDNF may exert cardioprotective effects in the acute phase of myocardial infarction. The occurrence of acute myocardial infarction is accompanied by pathological changes such as sympathetic hyperactivity, pro-inflammatory cytokine release, and activation of related signaling. The above pathological processes can lead to impaired energy metabolism in cardiomyocytes leading to apoptosis, cardiac fibrosis, and structural remodeling (33). BDNF is involved in cardiomyocyte protection and cardiac remodeling after myocardial infarction. Studies have shown that in the early stage of myocardial infarction, BDNF is increased in cardiomyocytes in the infarcted and peripheral regions. Elevated levels of BDNF can further activate BDNF-TrkB signaling, inhibit cardiomyocyte apoptosis, and exert a myocardial protective effect (34). Besides, after the occurrence of acute ischemic injury of the myocardium, the expression of BDNF in the central nervous system increases through neurofeedback. BDNF can enter the circulation through the blood-brain barrier and then reach the cardiovascular system, activating myocardial repair and angiogenic mechanisms (35). In addition, Increased physical activity after myocardial infarction promotes BDNF expression in the hypothalamus, and through BDNF binding to pro-myosin receptor kinase B activates ERK1/2 (the extracellular signal-regulated kinase 1/2) and AKT pathways, promoting macrophage survival (36). Macrophage-associated activity, on the other hand, is closely related to cardiac repair after myocardial infarction (37). It was also found that appropriate exercise during the recovery period of myocardial infarction can activate the BDNF-TrkB-FL-PI3K/Akt pathway to promote collateral angiogenesis and improve myocardial blood supply (36).

### 2.3 BDNF is a marker of structural remodeling and functional status of the heart in patients with heart failure

Heart failure is a clinical syndrome caused by structural and functional abnormalities of the heart, characterized by decreased cardiac output and inadequate perfusion of tissues and organs. Patients with heart failure have reduced exercise capacity, their quality of life is severely compromised, and they are often hospitalized due to associated factors that worsen their symptoms. BDNF is involved in endogenous myocardial repair and regulation of myocardial contractility in heart failure patients (38). The binding of BDNF and its receptor TrkB-T1 in cardiomyocytes activates calmodulin-dependent protein kinase II (calmodulin-dependent protein kinase II), which regulates calcium cycling and enhances myocardial contractility (39). Reduced levels of brain-derived neurotrophic factor, decreased expression of BDNF and its receptor, the pro-myosin-related kinase receptor (TrkB), in cardiomyocytes, and impaired energy metabolism in cardiac cells have been found in patients with heart failure (40). Another animal study found that the lack of BDNF in cardiomyocytes can lead to a series of pathological changes such as cardiomyocyte degeneration and death, left atrial appendage thrombosis, cardiac inflammation, functional decline, and metabolic disorders (41). BDNF is also involved in cardiac remodeling in heart failure patients. One study found that a reduction in circulating BDNF levels can cause adverse cardiac remodeling, affecting the functional status of patients and leading to increased mortality (42). Therefore, it has been suggested that BDNF is positively correlated with patients’ conditions (43). An observational clinical study showed that in ambulatory stable heart failure patients with LVEF <50%, those with lower serum BDNF concentrations had more severe cardiac remodeling and dysfunction and higher NT-proBNP levels (44). Compared with NT-proBNP, which is the most commonly used biomarker for the diagnosis and prognosis of heart failure patients, BDNF is also closely associated with the condition of heart failure patients. Therefore, in the future, BDNF may also be used as a circulating marker for the identification of cardiac remodeling and dysfunction in heart failure patients.

## 3 BDNF is involved in the process of cardiac rehabilitation

In cardiovascular diseases, BDNF is involved in cardioprotective and cardiac rehabilitation processes. In the acute phase, neural signals from the heart can be uploaded centrally, leading to an increase in BDNF expression and thus cardioprotection (35). During cardiac rehabilitation, exercise-induced changes in neuroplasticity increase circulating BDNF levels, which in turn exerts its biological effects to improve cardiac structure and function. Exercise can elevate the level of BDNF has also been confirmed by many scholars. BDNF is mainly released by the brain, crosses the blood-brain barrier enters the blood circulation, and then distributes to other tissues and organs to exert biological effects. It has been found that the brain releases three times more BDNF during exercise than at rest (45). One of the mechanisms by which exercise promotes BDNF release is the activation of astrocytes. Exercise promotes the proliferation and activation of astrocytes, which produce and release a variety of molecules including BDNF, nerve growth factor (NGF), glial cell line-derived neurotrophic factor (GDNF), and insulin-like growth factor1 (IGF-1) (46). Also, exercise activates astrocyte glycolysis to produce lactic acid. When the metabolic demand increases during exercise, glucose, which was originally stored in the form of glycogen, is rapidly degraded to lactate, and the generated lactate reaches the neuron through the monocarboxylic acid transporter to be used as an energy source, which is known as the astrocyte-neuron lactate shuttle (ANLS) (47). Lactate not involved in energy metabolism, together with that produced by muscle that enters the brain through the blood-brain barrier can promote BDNF release (48). The possible mechanism is that lactate induces the expression of PGC5α/FNDC1/BDNF signaling pathway through silent information regulator 1 (SIRT1) activation, which in turn increases the release of brain-derived neurotrophic factors (49). It has also been suggested that exercise promotes the cleavage of FNDC5 to irisin, which crosses the blood-brain barrier to induce BDNF production (50, 51). Another possible mechanism by which exercise promotes serum BDNF elevation is related to energy metabolism in skeletal muscle. In the process of functional activity, fat oxidation in skeletal muscle cells is enhanced, which can then promote the production of BDNF (52). Therefore, when malnutrition and exercise deficiency occur, abnormal skeletal muscle BDNF production mechanism and decreased serum BDNF levels can further lead to mitochondrial dysfunction in skeletal muscle and affect the efficacy of cardiac rehabilitation in patients with CVD (53).In summary, exercise elevates BDNF levels, which in turn play an important role in cardiovascular health and disease recovery; therefore, exercise-targeted BDNF may be a potential mechanism for its cardiac effects.

## 4 Future research directions in cardiac rehabilitation

### 4.1 Exercise prescription for cardiac rehabilitation

As a collection of repetitive physical activities, exercise is planned and organized. Exercise-based cardiac rehabilitation can benefit patients with cardiovascular disease by improving exercise capacity, reducing the risk of recurrence, and improving quality of life (54). A comprehensive exercise program for cardiac rehabilitation should include muscle strength training, exercise endurance training, and balance and flexibility activities (55). Aerobic exercise is one of the most commonly used forms of exercise in cardiac rehabilitation. Aerobic exercise improves cardiopulmonary adaptations and cardiovascular health (56). Cardiorespiratory health is associated with lower cardiovascular risk and good outcomes in cardiac patients. In recent years, resistance exercise has attracted the attention of scholars for its potential in preventing heart disease, improving clinical efficacy, and improving patients’ quality of life. Resistance training is a form of exercise with the main goal of increasing muscle strength and endurance. Resistance exercise has its advantages over intervention and drugs in terms of cost-effectiveness and continuity of efficacy (57). A systematic evaluation study showed that an exercise regimen that integrated resistance training with aerobic exercise yielded greater cardiovascular benefits than aerobic exercise alone (58). In addition to the above exercises,high-intensity interval training (HIIT) has also been recommended for patients with stable cardiovascular disease and has been suggested to provide additional benefits (59). A randomized controlled trial showed that high-intensity interval training (HIIT) was more effective than moderate-intensity continuous training (MICT) in improving physical indicators, aerobic capacity, and physical activity levels (60). Another safe and effective form of exercise that has been promoted by scholars in recent years is traditional Chinese exercise. Traditional Chinese exercises emphasize the unity of body and mind, breathing and exhaling, which can play a role in unblocking meridians and regulating qi and blood. By regulating sympathetic and parasympathetic nerve activity, traditional Chinese exercises such as tai chi can promote the establishment of coronary collateral circulation and improve cardiac function (61).

At present, various forms of exercise can be applied to cardiac rehabilitation, and related mechanism research is underway. It has been suggested that physical activity can promote “reverse remodeling” of the left ventricle in patients with coronary artery disease, improving its volume and ejection fraction (62). Related mechanistic studies show that improving vascular endothelial function, promoting vascular elastic adaptation, and reconstruction of coronary collateral circulation are potential physiological mechanisms of exercise-based cardiac rehabilitation effects on stable angina (63). In addition, clinical studies have found that early exercise can not only improve physical ability but also improve negative emotions and disease adaptation, especially in the young population of myocardial infarction patients (64). Others have suggested that up-regulation of peroxisome proliferator-activated receptor gamma coactivator 1 alpha expression, which in turn regulates cardiac energy metabolism (65), may be a potential mechanism for the cardiac effects of exercise. Behind the above mechanisms, BDNF may play a role. In the future, the influence of different exercise forms on BDNF needs further research, and relevant exercise prescriptions should be developed to achieve optimal rehabilitation results for patients.

### 4.2 The theoretical system of the brain-heart axis

BDNF is a key mediator connecting the brain-heart axis. The brain-heart axis refers to the complex bidirectional relationship between the central nervous system and the cardiovascular system. It is currently believed that the mechanisms involved in the brain-cardiac axis include the hypothalamic-pituitary-adrenal (HPA) axis, the autonomic nervous system (ANS), the systemic inflammatory response, and alterations in cardiovascular and cerebrovascular homeostasis (66). Blood circulation is the material basis for connecting the brain-heart axis. Analyzed in terms of hemodynamics, the heart serves as the power organ of blood circulation, providing oxygen and nutrients to the central nervous system. The brain is one of the most metabolically active organs in the human body, with 25% of the body's energy intake and 20% of its oxygen being consumed in the brain. Therefore, stable cerebral blood flow is of great significance to the structural function of the brain (67). Cardiovascular disease can lead to reduced cardiac output, inadequate cerebral perfusion, and reduced gray matter volume (higher metabolic demands on the brain's gray matter), which in turn leads to impaired brain function (68). The brain can likewise exert some influence on heart rate, myocardial contractility, etc. through blood circulation or neuronal signaling feedback mechanisms (69). In conclusion, the heart and the brain are closely related physiologically and can influence each other pathologically. For example, patients with heart failure may have secondary brain damage such as stroke, cognitive decline, and depression, while impaired brain function may exacerbate myocardial damage in patients with heart failure, leading to a vicious circle of worsening symptoms and disease progression (70). BDNF may play a mediating role in brain-heart interactions. BDNF is mainly synthesized and secreted by the central nervous system and can enter the cardiovascular system through the blood-brain barrier. In addition, BDNF can play a corresponding role in both the nervous system and the cardiovascular system. According to this analysis, BDNF may be a key protein connecting the brain-heart axis.

In addition to heart-related dysfunction, patients with cardiovascular diseases may be combined with brain function changes such as cognitive decline and psychological and emotional abnormalities such as depression. One Study has shown that cognitive decline is more rapid in patients with coronary artery disease than in healthy individuals, which is associated with lower circulating levels of brain-derived neurotrophic factor (BDNF) and insulin-like growth factor I, among others (71). Another study showed that among 225 patients with coronary artery disease, serum BDNF levels were lower in patients with comorbid depressive symptoms, suggesting a correlation between serum BDNF levels and depression in patients with coronary artery disease (72). Psychological problems, depression, and other comorbidities, once present, can further affect BDNF levels through the hypothalamic-pituitary-adrenal (HPA) axis, which in turn affects the patient's prognosis and leads to a vicious circle (73). Current studies have found that BDNF promotes neurogenesis, regulates synaptic plasticity, and is associated with a variety of neuropsychiatric disorders (7). Combined with recent studies, BDNF plays an important role in the cardiovascular system, so influencing BDNF through exercise to exert multiple biological effects can help to ameliorate physical and mental disorders in patients. In the future, relevant studies need to be conducted to construct a brain-heart axis theoretical system mediated by BDNF, and further improve patients’ brain-heart complications through exercise.

## 5 Summary and outlook

Cardiovascular diseases can have long-term adverse effects on patients. Short-term recuperation, long-term prevention, and continuous rehabilitation have become important principles in the management of cardiovascular disease. Cardiac rehabilitation plays an important role in reducing cardiac events, improving cardiac function, improving quality of life, and increasing social participation (74). Cardiac rehabilitation covers multidisciplinary programs such as exercise training, risk factor control, psychosocial assessment, and management. As an important intervention in cardiac rehabilitation, exercise plays an important role in the whole life cycle of patients with cardiovascular disease (75, 76). In the hospitalization phase, intensive care and treatment are the main focus, and appropriate exercise workouts are beneficial to the patient's recovery based on the assessment of risk. In the post-discharge phase, controlling risk factors, implementing exercise training, and addressing physical, mental, and social issues are the main components of cardiac rehabilitation. The role of exercise in improving cardiovascular health, reducing the risk of recurrence and acute cardiovascular events, and improving the overall health of patients cannot be replaced by traditional medications. The beneficial effects of exercise on the cardiovascular system may be closely related to BDNF, which is involved in the maintenance of vascular endothelial homeostasis and plays a role in controlling or reversing atherosclerosis, altering the course of coronary artery disease, and reducing the risk of adverse events such as sudden death and reinfarction. BDNF is also involved in myocardial protection and damage repair and plays an important role in improving physical and mental functions. Given the important role of BDNF in maintaining and promoting cardiovascular health, BDNF may become an important research target for cardiac rehabilitation in the future. Meanwhile, as a possible mediator of the brain-heart axis, the role of BDNF in brain-center interaction also needs to be further explored and studied.

## Data Availability

The original contributions presented in the study are included in the article/Supplementary Material, further inquiries can be directed to the corresponding author.
